# Getting there: Thyroid hormone receptor intracellular trafficking

**DOI:** 10.1016/j.jbc.2021.100677

**Published:** 2021-04-19

**Authors:** Lizabeth A. Allison

**Affiliations:** Department of Biology, William & Mary, Williamsburg, Virginia, USA

**Keywords:** thyroid hormone, nuclear receptor, nuclear translocation, acetylation, intracellular trafficking, NES, nuclear export signal, NLS, nuclear localization signal, RTH, resistance to thyroid hormone, TRE, thyroid hormone responsive element

## Abstract

A year ago, when I first contemplated writing this article, my intent was to provide a detailed review of the contributions of the diverse community of talented scientists in my lab to the nuclear receptor research field. In the throes of a deadly pandemic, political turmoil, and Black Lives Matter, however, I found myself compelled to tell a more personal story. While I will still cover milestones in our understanding of the intracellular trafficking of the thyroid hormone receptor, now these will be set against the backdrop of my path as a woman in STEM and on being intentionally inclusive. By sharing reflections on my journey, I hope to encourage young investigators to persist in their pursuit of a career in science.

Due to COVID-19 and the cancellation of ASBMB 2020, my acceptance of the 2020 Ruth Kirschstein Diversity in Science Award was deferred to 2021. I have now had a year to reflect on this humbling honor in the midst of chaotic times, filled with grief, fear, and division. My impact on the world at first glance seems akin to tiny ripples in a small pond, but as I think about the colleagues and students whose scientific careers I’ve encouraged and cultivated, I realize that those tiny ripples have set in motion forces of great consequence. If I am a ripple, my colleague, Dr Shantá D. Hinton is a surging wave.

What are the characteristics of a successful scientist? I can think of many words—intelligent, courageous, persistent, and full of initiative. Notably absent from my list are descriptors alluding to differences across socially constructed identities, such as race, ethnicity, nationality, religious affiliation, sexual orientation, gender identity, disability status, or socioeconomic background. It is abundantly clear that no scientific evidence exists to suggest that scientific potential and talent differentially segregate across these social identities. But if we are not part of the majority, “getting there” can be a daily obstacle course fraught with inequity and microaggression. Systemic racism takes its toll. Negative stereotypes about Black, Indigenous, and People of Color continue to be perpetuated. Sexist and anti-LBGTQ+ attitudes are pervasive. By sharing my unique path, I hope to encourage anyone starting out in science—who wonders if they belong—to persist.

## Getting started

**Figure 1:**
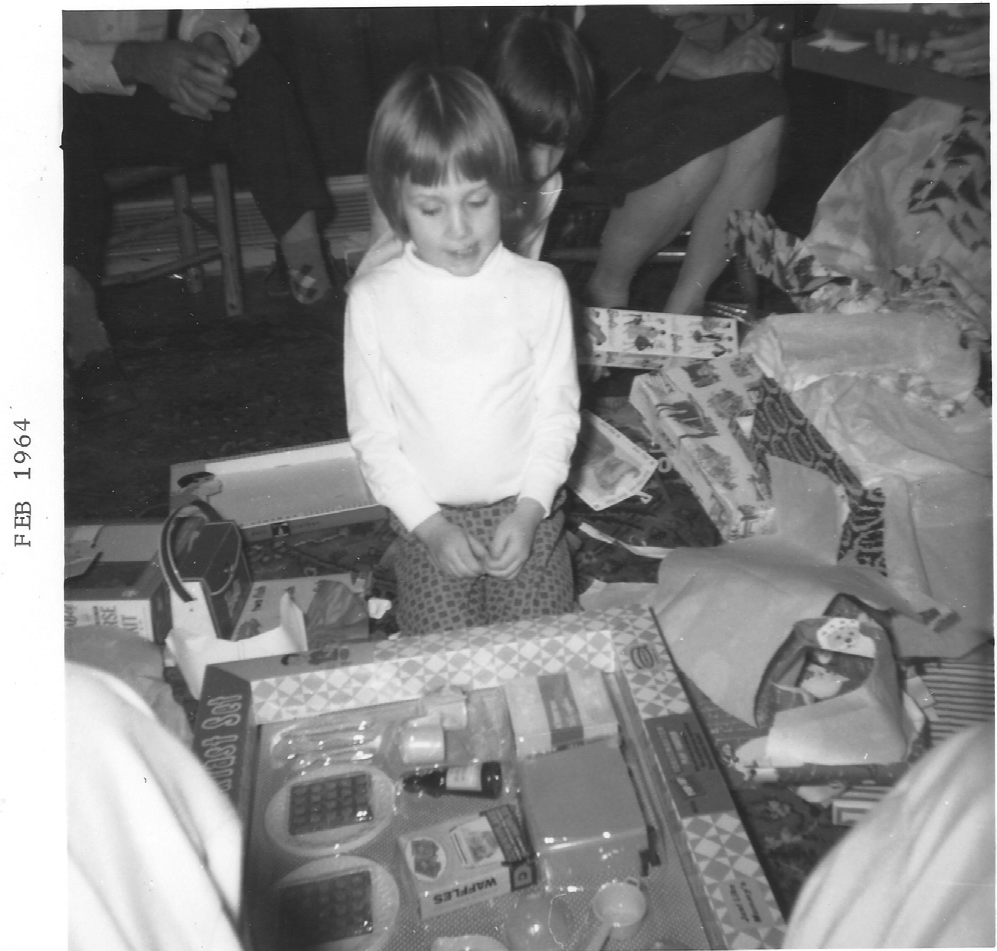
**The author at age 6 with her toy waffle breakfast set**.

The year I was born, Dr Martin Luther King, Jr wrote “True peace is not merely the absence of tension; it is the presence of justice” (1). Through their actions, my parents taught me about justice, kindness, dignity, and respect, but they also passed on a heavy dose of low self-esteem and humility to a fault. My older sister and I were raised in a very traditional household, until my parents divorced when I was 16. My father was a high school chemistry teacher and my mother a homemaker. My father’s view was that girls should be educated so that they would be good mothers in the future. Not surprisingly, rather than a chemistry set, my first memorable gift was a kitchen play set, complete with a toaster and plastic waffles (Fig. 1).

My early childhood experiences shaped important parts of me. Cooking, baking, and exploring the cuisine of different cultures remain a source of pleasure and comfort, providing a point of connection with others, a way of offering thanks, appreciation, or condolences when words may not suffice. Exploration and travel also figured prominently in my childhood. I grew up in Bellingham, Washington, sandwiched between Puget Sound and the North Cascades. When I wasn’t reading, I spent much of my time outside, catching frogs in our creek, hiking, biking, canoeing, swimming, and planning family trips. Money was tight, so those early trips were limited to where we could get by station wagon. My fascination with travel and exploration of the world around me shaped my ultimate research career, focusing on travels in the cellular world and the diversity of pathways into and out of the nucleus.

## From Fairbanks to Seattle to Christchurch

As a child and teenager, I felt most at home in school, although I didn’t know what I wanted to be when I grew up. On different days of the week I wanted to be a writer, a concert pianist, an Olympic athlete, an astrophysicist, an actress, and much later a biologist. I was the first woman in my immediate family to attend college, and as a pioneer in that way, it was only fitting to embark on my academic journey in Alaska, the last frontier. The University of Alaska-Fairbanks served as an ideal place to study biology as an undergraduate and then as a master’s student. When I started graduate school first in Alaska, and then as a PhD student at the University of Washington in the 1980s, it was a time when few women were high-ranking academic scientists. Without female role models, it was my male academic and research mentors, Drs L. Gerard Swartz and Gerald Shields, who saw a potential in me that I didn’t know I had and pointed me on the path to becoming a professor.

Driving around Alaska collecting black fly larvae from streams and analyzing their polytene chromosomes during the long, dark winters solidified my passion for research. I had assumed I would continue combining molecular biology with field work, but as a PhD student I found myself drawn to the intricacies of the molecular world and began exploring the intracellular trafficking of 5S ribosomal RNA in *Xenopus* oocytes in the lab of Dr Aimee Hayes Bakken.

This same research area—traffic control in cells—provided a launching pad for my independent career, starting in 1989 as a Lecturer and later a Senior Lecturer at the University of Canterbury in Christchurch, New Zealand. Bypassing traditional postdoctoral training made for a wobbly foundation, in which I always felt like I was playing catch-up, but I thrived on being in charge of a lab of my own, an aspect of academic life that appealed early on and that I still relish. As a bench-scientist in a spectacular country, I satisfied my love of the outdoors by volunteering as often as possible to assist colleagues in banding Little Blue Penguins or tracking wetas at night.

## Next stop: William & Mary

The next phase of my journey found me leaving New Zealand in 1997 to take a faculty position at William & Mary in Williamsburg, Virginia, where I continued my work on travels in the cellular world using the thyroid hormone receptors as a model system, earning tenure and promotion to full professor along the way. I have been fortunate over the years to work in my lab with an incredibly talented array of developing scientists from many different backgrounds and social identities, with over 150 undergraduates, 13 Master’s students, five PhD students, two postdoctoral fellows, and five visiting scientists. I thrive on the intellectual stimulation, innovation, and inspiration that comes from their diverse perspectives. As I relate some milestones in our understanding of thyroid hormone receptor intracellular trafficking, always underpinning the science are the people who made this work possible.

A member of the nuclear receptor superfamily, the thyroid hormone receptors, including the major subtypes TRα1, TRβ1, and TRβ2, are both intracellular receptors and transcription factors that act as both repressors and activators of their target genes in response to thyroid hormone. As such, they are able to bypass the complex second messenger signaling pathways and phosphorylation cascades of cell surface receptors. At the time I entered the nuclear receptor field, the long-standing dogma was that thyroid hormone receptors reside solely in the nucleus tightly bound to DNA. I began with a simple quest to identify nuclear localization signal (NLS) motifs in TRα1 and to determine with which importins it interacted, while traveling from its site of synthesis on cytosolic ribosomes, through the nuclear pore complexes into the nucleus. As is often the case in science, the quest turned out not to be so simple. In 2001, work begun by my last doctoral student in New Zealand and completed by my newly established team of undergraduates at William & Mary, revealed that TRα1 shuttles rapidly between the nucleus and cytoplasm (2). This finding launched my lab into a relatively unexplored aspect of thyroid hormone receptor function that has turned out to be a fascinating journey with many unexpected twists and turns.

Soon thereafter, another team of students showed that phosphorylation of TRα1 is compartment-specific and plays a role in nuclear retention, suggesting that multiple factors contribute to thyroid hormone receptor shuttling dynamics (3). In subsequent studies, we discovered that dominant negative thyroid hormone receptor mutants, including v-ErbA, the highly mutated viral oncogenic homolog of TRα1, localize to both nuclear and cytosolic compartments and display altered transport activity (2, 4, 5, 6, 7). Prior to our work on the oncoprotein v-ErbA, its dominant negative activity was attributed to competition with TRα1 for thyroid hormone responsive elements (TREs) and auxiliary factors involved in transcriptional regulation. Our studies defined a new mode of action of v-ErbA *via* subcellular mislocalization.

Undergraduates and graduate students in my lab, along with students and visiting faculty from Hampton University, a local HBCU, collaborated to show that the v-ErbA oncoprotein is highly mobile and traffics between the nucleus, cytoplasm, and aggresome. These findings led us to propose that disease-causing mutations target intracellular trafficking of the thyroid hormone receptors (4, 5, 6, 7, 8). Recent work spearheaded by a team of William & Mary undergraduates has shown that mutations in TRα1 that are associated with hepatocellular carcinoma, renal cell carcinoma, and thyroid cancer, lead to striking shifts toward a more cytoplasmic localization for many of the mutants and an increased tendency to form cytosolic and nuclear aggregates (https://www.kenzpub.com/journals/nurr/inpress/2020/101453/).

**Figure 2:**
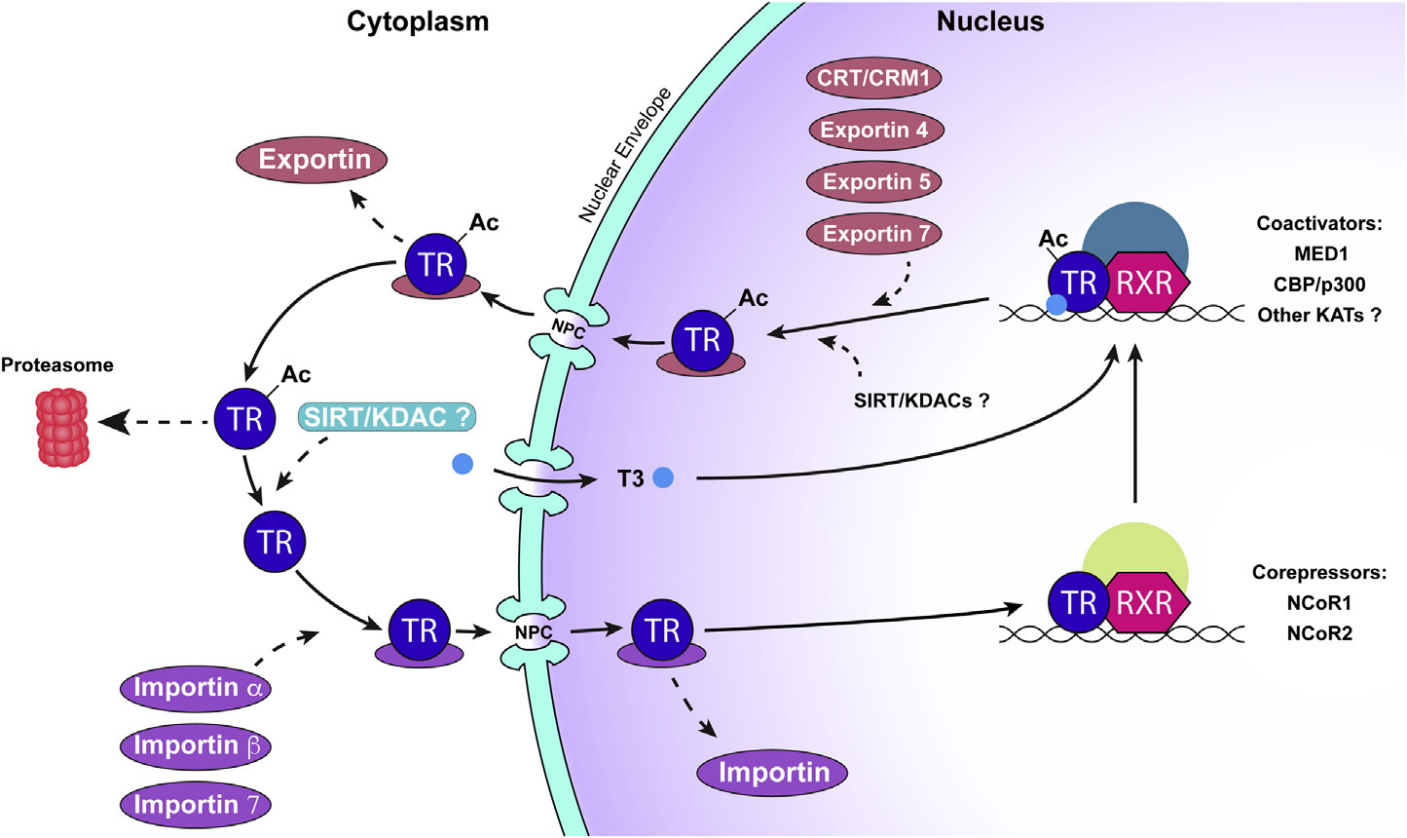
**Thyroid hormone receptor (TR) nucleocytoplasmic shuttling pathway.** The well-characterized pathway for TRα1 elucidated by our lab is depicted. TRα1 binds to specific importins in the cytoplasm, as indicated. The TRα1–importin complex passes through a nuclear pore complex (NPC) embedded in the nuclear envelope into the nucleus, where the complex is disassembled and TRα1 binds to target genes. TRα1 exits the nucleus through the nuclear pore complex (NPC) in association with specific exportins or the calreticulin (CRT)/CRM1 (exportin 1) complex. TRβ1 follows a similar nucleocytoplasmic shuttling pathway, but nuclear import is solely mediated by the importin α1/importin β1 complex. TR activation involves a multifaceted cascade of events that culminates in binding DNA and modulation of target gene expression. TRs often heterodimerize with the retinoid X receptor (RXR). Corepressors, such as N-CoR1 and N-CoR2, and histone deacetylases, are bound to TR in the absence of ligand. Upon thyroid hormone (T3) binding, TR undergoes a conformational change, resulting in binding of a new set of activator proteins, such as mediator subunit MED1 and CBP/p300, a lysine acetyltransferase (KAT). Nonacetylated TRα1 and TRβ1 are primarily localized to the nucleus and have reduced intranuclear mobility, while the acetylated receptors have greater intranuclear mobility and localize to the cytoplasm. Acetylated receptors may be targeted for proteasome-mediated degradation or deacetylated by a sirtuin (SIRT) or lysine deacetylase (KDAC). The cellular compartment for TR deacetylation remains unknown (as indicated by the *question marks*).

Upon discovery that thyroid hormone receptors follow an elaborate pathway within cells, we shifted our focus from nuclear import alone to characterizing their nuclear export pathway, completing a comprehensive, systematic characterization of their multiple NLSs and nuclear export signals (NESs), and the multiple transport pathways that mediate nuclear entry and exit (Fig. 2). Mutagenesis studies on an NES in helix 12 of the ligand-binding domain pointed to the intriguing possibility that altered shuttling of TRα1 may be a contributing factor in Resistance to Thyroid Hormone (RTH) syndrome, providing further support for our model that intracellular mislocalization of thyroid hormone receptors is an important factor to consider in pathogenesis (https://www.kenzpub.com/journals/nurr/inpress/2020/101453/) (8, 9, 10, 11, 12, 13, 14).

Our attention has now shifted to examining the multifaceted factors impacting nuclear retention and intranuclear mobility of the thyroid hormone receptors, including interactions with Mediator (15), and posttranslational modification (9). Another team of master’s students and undergraduates demonstrated that mediator subunits MED1, and to a lesser extent MED13, promote nuclear retention of TRα1 and TRβ1 by altering intranuclear dynamics, highlighting how helix 12 in the ligand-binding domain of the thyroid hormone receptors modulates both gene transactivation and nuclear export (15). Given the proximity of known thyroid hormone receptor acetylation sites to NLS motifs, we next investigated their role in regulating intracellular localization. Here, the driving force behind the “we” was Cyril Anyetei-Anum, first an undergraduate in my lab and then a master’s student, who found his life’s passion and is now pursuing a PhD at the University of North Carolina at Chapel Hill. Cyril’s work revealed that while TRα1 and TRβ1 nonacetylation mimics display wild-type distributions, the acetylation mimics were significantly more cytosolic. In addition, fluorescence recovery after photobleaching analysis showed wild-type intranuclear dynamics of acetylation mimic thyroid hormone receptors, whereas the nonacetylation mimics had significantly reduced mobility and transcriptional activity, further suggesting that nonacetylation correlates with nuclear retention, while acetylation promotes cytosolic localization. Based on these findings, Cyril proposed a novel model in which acetylation acts as a regulatory switch for nucleocytoplasmic shuttling of the thyroid hormone receptors (see Fig. 2). Our ongoing studies led by a new team of undergraduates and master’s students are investigating the underpinning mechanism of this acetylation switch. Through long hours at the bench, together, we have increased our knowledge of how thyroid hormone receptors “get there” (to the nucleus), but the biological significance of their shuttling remains a puzzle to solve. These discoveries are rewarding and exhilarating, but equally rewarding is being in a position to create opportunities for young investigators to succeed in science.

## On belonging in science

Early on in my career, I become aware that I was often viewed first as a blonde-haired, blue-eyed woman and second, as a scientist; and the bar was noticeably higher when my accomplishments were judged. It is easy to feel like an outsider, or an imposter, when spoken or upspoken, “not bad for a girl” suffuses the air. I did not become preoccupied with how I was viewed or let this discourage me. Rather, this awareness simply added to my motivation to flourish as a scientist and to maintain my own dynamic, externally funded lab. In this way, I can maintain a welcoming, inclusive space that enables each student or colleague to access the resources they need in order to thrive as scientists.

As the Chair of the Biology Department, from 2009 to 2014, and the first woman to hold this leadership position, I dove in with energy and a vision for change. In my first year, I used my connections to orchestrate the recruitment of the first faculty member of color in the natural sciences at William & Mary. Filled with my usual optimism and positivity, I was blindsided by the personal costs involved in forcing change. When I had to acknowledge that there are colleagues who subconsciously or consciously struggle to affirm that people of darker pigmentation are intelligent and qualified scientific colleagues, a part of my spirit was crushed. Having always been comfortable in my interactions with people from diverse backgrounds, I found myself confused and disappointed. As I stood by my new colleague, a gifted scientist and rising star in her field, I shared her frustration at having grant after grant application rejected for years before she finally succeeded. There was no denying that racial bias by funding panels (16) was part of the story. There was anguish and anger along the way, but we persisted and the payoff was tremendous. The message to young, underrepresented minority investigators: you belong in science.

I have mentored students that would have succeeded in any lab, along with students that needed extra encouragement to show their true potential. It is these latter students that most benefit from my mentorship. These interactions are not one-sided. I, in turn, have had to grow as a mentor, adjusting my usual quiet, understated style to one with a stronger voice. There are conversations that no one should need to have. Like having to bluntly tell aspiring young Black investigators in my lab that to be validated as scientists, to have their voices heard, to be perceived as intellectual equals, they would have to work at least twice as hard as their white peers. Although unfair, this is the reality our nation has propagated. There is no room for mistakes in the lab or out on the street.

The bottom line is that, as scientists, whatever our backgrounds or social identities, we all take joy in performing experiments and share the thrill of making novel discoveries. There is great satisfaction for me in cultivating talent and providing encouragement and opportunities for all scientists in the making, without arbitrary filters such as skin color, gender identity, or neurodiversity. Over the years, my lab has included people from backgrounds that are traditionally underrepresented in science and those from backgrounds that are traditionally well represented. We have conversations together, we do better science together, we solve problems together from diverse perspectives, and we share meals together, or at least we did pre-COVID-19. Our conversations may not always be calm and graceful, but as long as we sit down together and keep talking, progress can be made. Our diversity enriches and stretches us. We are stronger and more creative. And, we have each other’s backs, as we navigate the daily obstacle course together as a community of scientists.

## Final thoughts

I was a puzzle to my father until the end of his life in 2004 as he grappled with a daughter who was more like his version of a son. He was proud of me but confused by me. When I eventually married my husband 24 years ago, my father expressed concern about the impending loss to the scientific community, since he assumed that I would quit my career once married. He would be pleased that I am still employed, and would be proud of his grandson, a senior in high school who is taking AP Chemistry. Sadly, my mother died before my son was born, never seeing what she would have viewed as my greatest achievement.

It is exhausting to live each day with fear, divisiveness, and incivility. So, in 2021, I will move forward with hope, finding joy in science and cultivating talent. I like to point out to my students that when it comes to identity, I am “mixed species”—a combination of *Homo sapiens*, Neanderthal, and Denisovan DNA. Near the end of each class I have taught, whether molecular genetics or introductory biology, I always read one of my favorite poems, *Human Family* by Maya Angelou, which ends with the refrain: “I note the obvious differences/between each sort and type/but we are more alike my friends,/than we are unalike.”

## Conflict of interest

The author declares that they have no conflicts of interest with the contents of this article.
